# Leaf epicuticular wax and hormone‐mediated resistance to *Alternaria brassicicola* in broccoli

**DOI:** 10.1111/ppl.70172

**Published:** 2025-04-04

**Authors:** Sunil S. Gangurde, Navjot Kaur, Baozhu Guo, Bhabesh Dutta

**Affiliations:** ^1^ Department of Plant Pathology University of Georgia Tifton Georgia U.S.A.; ^2^ U.S. Department of Agriculture, Agricultural Research Service Crop Genetics and Breeding Research Unit Tifton Georgia U.S.A.

## Abstract

Alternaria leaf spot disease is a foliar disease of brassica crops, including broccoli, caused by *Alternaria brassicicola.* This disease is a serious problem causing unmarketable yields and millions of dollars in losses in broccoli and other crucifers across the globe. During the pathogenicity and whole plant inoculation assays, we observed significantly higher disease severity in the older leaves at the bottom, as compared to the younger leaves at the top. The symptoms seemed to appear first in the lower and older leaves and gradually progressed upwards to the younger leaves, ultimately reaching the broccoli head. Epicuticular wax analysis showed a significantly higher amount of wax deposition on the younger leaves at the top as compared to the older leaves at the bottom. Further regression analysis showed a negative correlation between wax per unit area and disease severity. Differential gene expression analysis showed upregulation of key wax biosynthesis genes, namely 3‐ketoacyl‐CoA synthase (*Bol018447*), alkane hydroxylase CYP96A15 (*Bol016302*) and O‐acyltransferase WSD1 (*Bol024738*) in the younger leaves. The abscisic acid, salicylic acid and jasmonic acid were differentially accumulated in the older leaves as compared to the younger inoculated leaves in response to *A. brassicicola* inoculation. Transcriptome analysis uncovered large transcriptome reprogramming in the older leaves as compared to the younger leaves. Senescence‐associated genes such as senescence regulator S40 (*BolC7t42093H*), stress up‐regulated Nod 19 (*BolC2t12223H*), and late embryogenesis abundant protein (*BolC8t47646H*) were upregulated in the older leaves. These findings suggest that the older bottom leaves of broccoli are highly susceptible to *A. brassicicola*, potentially associated with lower wax deposition and increased modulation in senescence hormones.

## 1 INTRODUCTION

Broccoli (*Brassica oleracea* L. var. *italica*) is grown in both, the fall and spring growing seasons in Georgia, USA (Coolong et al., 2016). Broccoli production has been negatively impacted by several pests and diseases, and one such disease is Alternaria leaf blight and head rot (ABHR) disease caused by an *Alternaria* species complex, which includes *Alternaria brassicicola* (Schwein.), *A. brassicae* [Berk.] Sacc. and *A. raphani* (Groves & Skolko; syn. *A. japonica*) Nowakowska et al., 2019; Nowicki et al., 2012). Routine outbreaks of ABHR occur in broccoli and in other brassicas across the eastern United States and have been routinely causing losses in yield and quality (Nieto‐Lopez et al., 2023; Kreis et al., 2016). Although different species of Alternaria have been isolated from the symptomatic plants, *A. brassicicola* has been the predominant species, which is routinely isolated from ABHR outbreaks in commercial broccoli fields (Kreis et al., 2016; Nieto‐Lopez et al., 2023).

The pathogen can affect broccoli at any growth stage, and symptoms include leaf spots, damping‐off in seedlings, and black circular spots on stems, leaves, and heads of matured plants (Nowicki et al., 2012). This pathogen was also associated with contaminated commercial broccoli seeds and in some cases aggressive and azoxystrobin (a Quinone‐outside inhibitor (QoI)) fungicide‐resistant *A. brassicicola* isolates were also recovered (Kaur et al., 2024). Apart from surviving in seeds, the pathogen can also survive on infected crop debris from year to year (Humpherson‐Jones, 1989).

Host resistance, mediated by either plant physical structures or by the production of secondary metabolites like phytoalexins, form the first line of defense against any invading pathogens (Boutrot and Zipfel, 2017; Sajjo et al., 2018). Physical structures such as trichomes, spines, thorns, hairs, and cuticular wax that cover plant surfaces act as barriers to pathogen penetration, and are widely known in different pathosystems (Lewandowska et al., 2020; Aragon et al., 2017; Favaro et al., 2020). Previous reports have suggested that cuticular wax biosynthetic genes were significantly induced by drought stress in broccoli. Authors reported differential expression of six cuticular wax biosynthesis genes: *LACS1*, *KCS1*, *KCR1*, *ECR*, *CER3* and *MAH1* in bloomed as compared to bloomless broccoli plants (Lee et al., 2015). In another brassica crop, cabbage (*Brassica oleracea* var. capitata), researchers investigated the expression of epicuticular wax biosynthetic genes at different developmental stages and reported that expression increased initially with leaf development and then decreased in cabbage lines with less epicuticular wax content (Laila et al., 2017). Despite these limited studies that investigated the role of epicuticular wax on abiotic stress in broccoli, none of the studies investigated the role that epicuticular wax might play in *A. brassicicola* infection in broccoli. We hypothesized that epicuticular wax plays a significant role in the host susceptibility against *A. brassicicola* infection in broccoli.

Genes related to systemic acquired resistance (SAR) like non‐expressor of pathogen‐related gene 1 (NPR1), as well as pathogenesis‐related (PR) genes, can play a significant role in inducing resistance against a wide range of pathogens (Pieterse et al., 1998). NPR1 plays a crucial role in inducing a defense signaling network via hormones such as salicylic acid (SA) and jasmonic acid (JA)/ethylene responses (Cao et al., 1998). When triggered by biotrophic pathogens, the SA pathway is involved in SAR, which can induce the expression of PR genes such as *PR1*, *PR2*, and *PR5* that further aid in providing resistance against a wide range of pathogens, although not as strong as effector‐mediated resistance. In contrast, the JA pathway is mainly activated by necrotrophic pathogens, resulting in locally acquired resistance via the upregulation of *PR3*, *PR4*, and *PR12* genes (Ali et al., 2018; Nahar et al. 2011).

Another aspect of resistance is related to plant maturity, which is also known as age‐related resistance or susceptibility (Kus et al., 2002). Earlier age‐related resistance was reported in broccoli against downy mildew caused by *Peronospora parasitica*, where matured plants (5–6 true leaf stage) showed higher levels of resistance than younger plants (2–3 true leaf stage; Coelho and Monteiro, 2003). In *Brassica napus*, it is also reported that the quantitative disease resistance (QDR) is determined by the lifestyle (biotrophic, hemibiotrophic, necrotrophic etc.) of the pathogen (Jacott et al., 2024). Different leaf growth stages can also impact susceptibility to invading pathogens due to age‐related resistance (Hu and Yang, 2019). Earlier reports in cabbage and broccoli indicated that the younger and upper leaves were less susceptible to downy mildew infection than the older and lower leaves (Coelho et al., 2009). Apart from these limited studies, there are no reports on age‐related resistance/susceptibility of broccoli against *A. brassicicola* infection. Apart from these aspects, we also investigated the differences in plant hormones and their associated genes, and other transcript level changes in broccoli leaves at different maturity stages against *A. brassicicola* infection.

## 2 MATERIALS AND METHODS

### 2.1 Plant and fungal material

Certified broccoli seeds (cv. Eastern Crown) were procured from a reputed seed company (Sakata Seed America Inc., Woodland, CA). The seeds were sown in seedling trays and grown for two weeks in a greenhouse under controlled conditions of 25–28°C and 70–90% relative humidity with a light:dark cycle of 12:12 h. Osmocote smart‐release plant food (The Scotts Company, Marysville, Ohio) was used for periodic fertilization. Later, the seedlings were transplanted into large size plastic pots. All plants were grown and assayed when the plants reached a 6‐true leaf stage. A well‐characterized aggressive isolate of *A. brassicicola* ‘F5A8’ collected from broccoli fields in Georgia was used in all experiments. The fungal culture was grown on full‐strength potato dextrose agar with 12 h of light and 12 h of dark at 25°C for seven days. For all pathogenicity assays, the concentration of the spore suspension was measured using a hemocytometer (Neubauer, PA) and adjusted to 1 × 10^5^ conidia milliliter^−1^. For control treatments, sterilized distilled water was used.

### 2.2 Pathogenicity assays

Three types of pathogenicity assays were performed at various leaf growth stages of broccoli to identify susceptible growth stages: an in vitro detached leaf pathogenicity assay; a pathogenicity assay on intact broccoli plants and a pathogenicity assay on wax and non‐wax leaf surfaces of top and bottom leaves of broccoli plants.

#### 2.2.1 In vitro detached leaf assay

**FIGURE 1:**
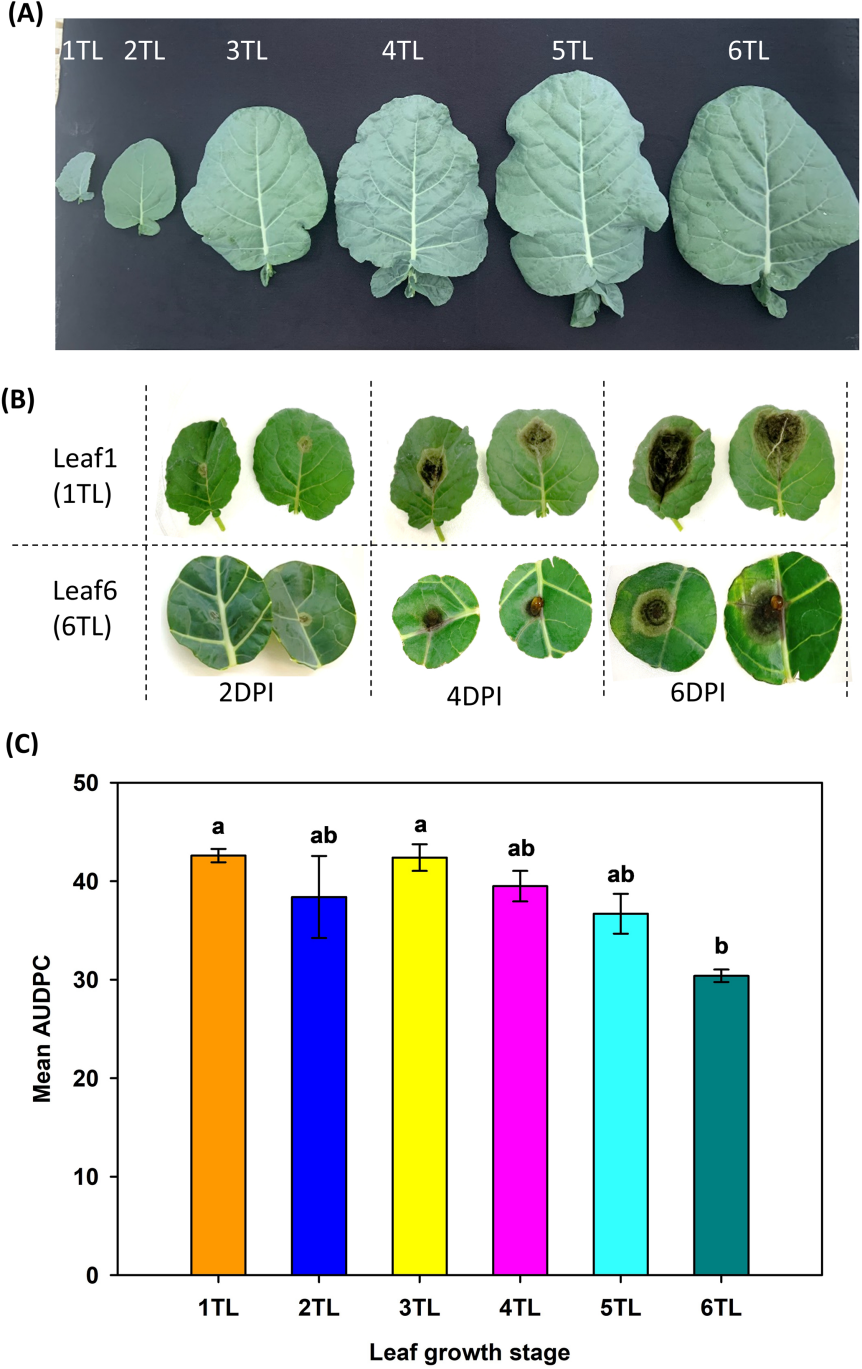
**Age‐related susceptibility of broccoli leaves against Alternaria brassicicola**. Pathogenicity assay on detached broccoli leaves at various growth stages, from first true leaf (1TL) stage to 6th true leaf (6TL) stage was performed. The panel (A) represents a pictorial representation of each fully expanded true leaf stage annotated as (1TL: first true leaf; 2TL: second true leaf stage; 3TL: third true leaf stage; 4TL: fourth true leaf stage; 5TL: fifth true leaf stage; and 6TL: sixth true leaf stage); (B) Phenotypically visible variation in the lesion sizes at 2, 4 and 6 DPI for two growth stages 1TL (oldest) and 6TL (youngest). (C) Mean area under disease progress curve (AUDPC) at each growth stage calculated based on lesion area (mm^2^) at 0, 2, 4 and 6 DPI. Means on the bars followed by the same letters are not significantly different according to Tukey's honest significant difference (*p* < 0.05).

Among three inoculation methods (cotyledon, detached leaf, and seedling inoculation), detached leaf inoculation was found to be most suitable for screening the pathogens in *Brassica rapa* (Doullah et al., 2006). The detached leaf assay was conducted on broccoli leaves at different leaf growth stages: first true leaf (1TL, bottom, oldest), 2nd true leaf (2TL), 3rd true leaf (3TL), 4th true leaf (4TL), 5th true leaf (5TL) and 6th true leaf (6TL, top, youngest) where true leaf means a fully expanded leaf (Figure 1A). Broccoli leaves were harvested by cutting leaf discs of a diameter of 24 mm using a sterilized cork‐borer. Two such leaf discs were placed in sterilized petri plates with moist Whatman no. 1 filter paper. The leaf discs were inoculated with 10 μL spore suspension of a concentration of 1 × 10^5^ conidia ml^−1^. The vertical and horizontal diameters of the lesions were recorded at two days post‐inoculation (DPI), 4 DPI and 6 DPI, and the average of the two diameters (*d*) was used to calculate the lesion area (mm^2^) using the formula: *π*(*d*/2)^2^. The Area Under the Disease Progress Curve (AUDPC) was calculated using the lesion area recorded at each DPI (mm^2^; Simko and Piepho, 2012). Six replicates per treatment aka. leaf growth stages, were used and a total of two independent experiments were conducted. ANOVA was used for AUDPC and Tukey's HSD test was used to determine the mean separation for the different leaf growth stages, while error bars represent the standard error of the means.

#### 2.2.2 Pathogenicity inoculation assay using intact broccoli plants

To determine the susceptibility of different leaf growth stages in intact broccoli plants, a whole plant inoculation pathogenicity assay was conducted. Intact broccoli plants were inoculated at their heading stage by spraying a spore suspension of 1 × 10^5^ conidia ml^−1^ until run off. The plants were sprayed with sterilized distilled water served as a negative control. Both control and inoculated plants were incubated at 12 h of light and 12 h of dark with 95% humidity for three days. Inoculated leaves were rated for percent disease severity, where a disease severity rating scale of 0 (no necrotic lesions observed) to 100% (entire leaf area is covered with necrotic lesions) was used at 0, 5, 10 and 15 DPI. The disease severity rating data was recorded for six true leaves from top (6TL) to bottom (1TL). The AUDPC was calculated for each true leaf using three disease severity ratings (Simko and Piepho, 2012). Four replicates per treatment (control or inoculated) were included in the experiment, and two independent experiments were conducted. ANOVA was determined for AUDPC and Tukey's HSD test was used to determine the mean separation for different leaf growth stages and treatment. Error bars represent standard error of the means.

#### 2.2.3 Pathogenicity assay for top (younger) and bottom (older) leaves with and without epicuticular wax

To test the effect of leaf epicuticular wax on the pathogenicity of *A. brassicicola*, a detached leaf assay was performed on the wax and non‐wax leaf surfaces of younger (6TL) and older (1TL) leaves. The wax of the right side from the middle vein of the leaf was wiped from both leaves using sterilized cotton plugs. While the wax layer of the left side of the middle vein was not disturbed for both of the leaves. Both wax and non‐wax sides of the leaves were inoculated with 10 μL spore suspension of *A. brassicicola* with a spore concentration of 1 × 10^5^ conidia ml^−1^ while sterilized water was used as control. The lesion diameters on the detached leaf tissues were measured at 2, 4 and 6DPI, and AUDPC was calculated. ANOVA was determined for AUDPC and Tukey's HSD test was used to determine the mean separation for different leaf growth stages and wax and non‐wax leaves, while error bars represent the standard error of the means.

### 2.3 Epicuticular wax determination and regression analysis with disease severity

The epicuticular wax content on leaves of various growth stages of broccoli plants was determined to compare the percentage of wax content and its role to act as a physical barrier for disease suppression. It was done by evaluating regression analysis between AUDPC and epicuticular wax percentage for each leaf at different growth stages. The epicuticular wax on the broccoli leaves was extracted using a chloroform extraction method and quantified as described in Munaiz et al., (2020). Briefly, six aluminum leaf pans were pre‐weighed (mg) for six true leaves and were labeled carefully. Each true leaf was detached from the main stem of the broccoli plant and added to the respective pans without disturbing the wax layer on the leaf surface. Further final weight was recorded for each leaf together with a pan. The leaf tissues were submerged in 250 mL of HPLC grade chloroform (Fisher# C606‐6) for 1 minute. Later, leaf tissues were removed, and the extracts were allowed to evaporate under the airflow supreme fume hood (Kewaunee Scientific Equipment Corp.) until complete evaporation of chloroform from the aluminum pan. Finally, the aluminum pans with the extracts were reweighed and the total wax content was calculated using the formula [(Final weight of empty pan after the chloroform has been evaporated‐ Initial weight of empty pan)/(weight of pan with leaf tissue‐weight of empty pan)] *100. The wax percentage of each true leaf was calculated. Three replicates per leaf were used in a single experiment, and the experiment was repeated independently. ANOVA was used to determine the percentage of the epicuticular wax content and a Tukey's HSD test was used to separate means.

The relationship between percent epicuticular wax content and AUDPC calculated from whole plant inoculations was determined using a regresssion analysis in R (version 4.3.1). The regression analysis was based on means of three replicates from Exp‐1.

### 2.4 Mining of epicuticular wax biosynthesis genes, *NPR1* and *PR* genes in the broccoli genome

During the wax determination experiment, a significant difference in wax content in different leaf growth stages was observed, so it led to our hypothesis that the wax genes were differentially expressed in different leaf growth stages of broccoli. To validate this hypothesis, wax biosynthesis genes from *Arabidopsis thaliana* with their accession numbers were used to find homologs of wax biosynthesis genes in the broccoli genome (GCA_900416815.2). A total of 20 wax biosynthesis genes were identified in the broccoli genome using the query sequences from *A. thaliana* (Supplementary Table 1). The CDS sequences for all these genes were used for designing the primers using the primer3 online tool with default parameters (https://primer3.ut.ee/). Primer sequences for wax biosynthesis genes are provided in Supplementary Table 2. To normalize the expression of genes, a total of three housekeeping genes namely *actin1*, *actin2* and *actin3* were tested for stable expression across different leaf growth stages. Housekeeping gene *actin2* showed the most stable expression across leaf growth stages. Expression values of the wax biosynthesis genes were normalized using *actin2*, and normalized expression values were log‐transformed and used for plotting the heat map to visualize differential gene expressions. Similarly, gene sequences for *NPR1* and *PR* genes (*PR1, PR4, PR5, PR6* and *PR12*) were retrieved from the broccoli genome and primers were designed using CDS sequences in the primer 3 software (Supplementary Table 3).

### 2.5 Total RNA extraction, cDNA synthesis and qRT‐PCR expression analysis

Three tissues including younger leaf (6TL), older leaf (1TL) and head from 120–125 days old broccoli plants were targeted for expression analysis of the wax biosynthesis genes. Total RNA was extracted from 60 mg leaf tissue using the Qiagen's RNeasy Plant Mini Kit as per the manufacturers protocol (Qiagen). The Qubit RNA high sensitivity assay kits were used for easy and accurate RNA quantification. The genomic DNA in the samples was removed using RNase‐free DNase enzyme (Invitrogen). The final concentration was diluted as per the requirement for cDNA synthesis using nuclease‐free water. For cDNA synthesis, a commercial Applied Biosystem's High‐Capacity cDNA Reverse Transcription Kit (Thermo Fisher Scientific) was used according to the manufacturer's instructions. Initially gene specific primers were amplified on a normal thermocycler using gradient PCR to standardize the annealing temperatures. Only the amplified primers were further used in the qRT‐PCR for expression analysis. The PCR conditions for real time PCR were as follows: 95°C for 10 min, 40 cycles at 95°C for 20 seconds, 58°C for 20 seconds and 72°C for 25 seconds. Three biological replicates were used for each sample. Three technical replicates were used for each biological replicate. Relative gene expression was calculated using the 2‐∆∆CT method (Livak and Schmittgen, 2001).

### 2.6 Analysis of plant hormones from top (younger) and bottom (older) broccoli leaves upon *A. brassicicola* inoculation

Along with the physical barrier, such as epicuticular wax, which can play a crucial role in defense mechanisms, we also hypothesized that plant hormones could also play an important role in inducing defense signaling pathways in the upper canopy of broccoli plants. To evaluate the role of plant hormones in defense signaling pathways, we analyzed the plant hormone profile of different leaf growth stages of broccoli. Plant hormones in the youngest leaf (6TL), oldest leaf (1TL) and the broccoli head were determined upon *A. brassicicola* inoculation. At head stage (120–125 days old plant), the whole plant was inoculated with a highly aggressive isolate of *A. brassicicola* as described previously in the whole plant pathogenicity assay. The leaves of control and inoculated plants were collected from 1TL or 6TL and a head at 10 and 20 DPI, and mean concentrations of three plant hormones, abscisic acid, salicylic acid and jasmonic acid were determined as described below.

Tissue samples from the young leaf (6TL), older leaf (1TL) and head from inoculated and control plants at 10 and 20DAI were selected for hormone analysis. Hormone analysis was carried out at the Creative Proteomics, Shirley, New York, United States. The sample preparation in brief: tissue samples were processed using cold methanol: acetonitrile (50:50, v/v) spiked with deuterium‐labeled internal standards. The tissue samples were disrupted using the Tissue Lyser II (Qiagen). After centrifugation at 16 000 g, the supernatants were collected, and the extraction of the pellet was repeated one more time. The supernatants were pooled and dried down using a speed‐vac. The pellets were re‐dissolved in 15% methanol and run using a LC–MS MRM (Multiple Reaction Monitoring) targeted assay. Briefly LC separation was done on a ZORBAX Eclipse Plus C18 column (2.1 mm × 100 mm, Agilent) flowing at 0.45 mL min^−1^. The gradient of the mobile phases A (0.1% acetic acid) and B (0.1% acetic acid/90% acetonitrile) was as follows: 5% B for 1 min, to 60% B in 4 min, to 100% B in 2 min, hold at 100% B for 3 min, to 5% B in 0.5 min. The Shimadzu LC system was interfaced with a Sciex QTRAP 6500+ mass spectrometer equipped with a TurboIon Spray (TIS) electrospray ion source. The instrument was set up to acquire negative and positive ion modes. The Analyst software version 1.6.3 was used to control sample acquisition and data analysis. The hormones were detected using MRM transitions that were optimized using standards. For quantification, an external standard curve was prepared using a series of standard samples containing different concentrations of unlabeled hormones and fixed concentrations of the deuterium‐labeled standard mixtures. ANOVA was used to determine different hormone concentrations separately and the mean separation was determined using the Tukey's honest significant difference (*p* < 0.05) and error bars represent the standard deviation of the means.

### 2.7 Transcriptome analysis

Transcriptome analysis was carried out from control and inoculated older (1TL) or younger (6TL) leaves of broccoli inoculated with *A. brassicicola* or the mock control with sterile water. Total RNA was extracted using Qiagen's RNeasy Plant Mini Kit (Qiagen) from 1TL and 6TL of control and inoculated plants at 10 DPI. Three biological replicates and three technical replicates for each tissue at each time point were used for RNA‐Seq analysis. The cDNA libraries were prepared using the mRNA‐Seq Sample Prep kit (Illumina Inc.) following the manufacturer's instructions. Paired‐end sequencing was carried out on the Illumina Hi‐Seq 2500 platform and raw reads of 100 bp were generated. Filtered reads were obtained after running the quality control (QC) using NGS‐QC box. Sequence reads were trimmed to remove possible adapter sequences and nucleotides with poor quality using Trimmomatic v.0.36. The trimmed reads were mapped to the *Brassica oleracea* (Var. HDEM) reference genome available on ENSEMBL using the STAR aligner v.2.5.2 and bam files were generated.

Unique gene hit counts were calculated by using feature Counts from the Subread package v.1.5.2. Only unique reads that mapped within exon regions were counted. Using DESeq2, a comparison of gene expression between the customer‐defined groups of the samples was performed. The Wald test was used to generate *p*‐values and log2 fold changes. Genes with a *p*‐value <0.05 and absolute log2 fold change >1 were called as differentially expressed genes for each comparison. The top 30 genes were sorted by their adjusted *p*‐values and used for visualization on a heat map to identify co‐regulated genes across the treatment conditions. Volcano plots were also developed by plotting the log2 fold changes of each gene on the x‐axis and the log10 of its *p*‐value was located on the y‐axis. Genes with a *p*‐value <0.05 and a log2 fold change greater than 1 were considered as upregulated genes and indicated by red dots. Genes with a *p*‐value <0.05 and a log2 fold change less than −1 were considered as downregulated genes and indicated by blue dots.

## 3 RESULTS

### 3.1 Variation in the age‐related susceptibility across different leaf growth stages of broccoli

The susceptibility of leaves at different growth stages to *A. brassicicola* infection was assessed in a detached leaf assay (Figure 1A). A significant variation in the lesion development across the different leaf growth stages of broccoli was observed. The lesion developed on the leaf at 2 DPI, which started initially as a small brown spot with a yellow halo that gradually became dark brown to black, and turned into a circular necrotic lesion by 6 DPI covering 90% of the leaf area (Figure 1B). The lesion area (mm^2^) and the associated AUDPC increased with increasing incubation period (DPI). There was a positive correlation between the lesion area (mm^2^) and incubation‐period (DPI) on each of the true leaf stages investigated (*p* > 2 × 10^−16^; *R* ^*2*^ = 0.851; y = −4.9 + 3.7x). The AUDPC values were significantly higher for the bottom‐most older leaf (1TL) compared to the youngest top‐leaf (6 TL; Figure 1B; 1C).

**FIGURE 2:**
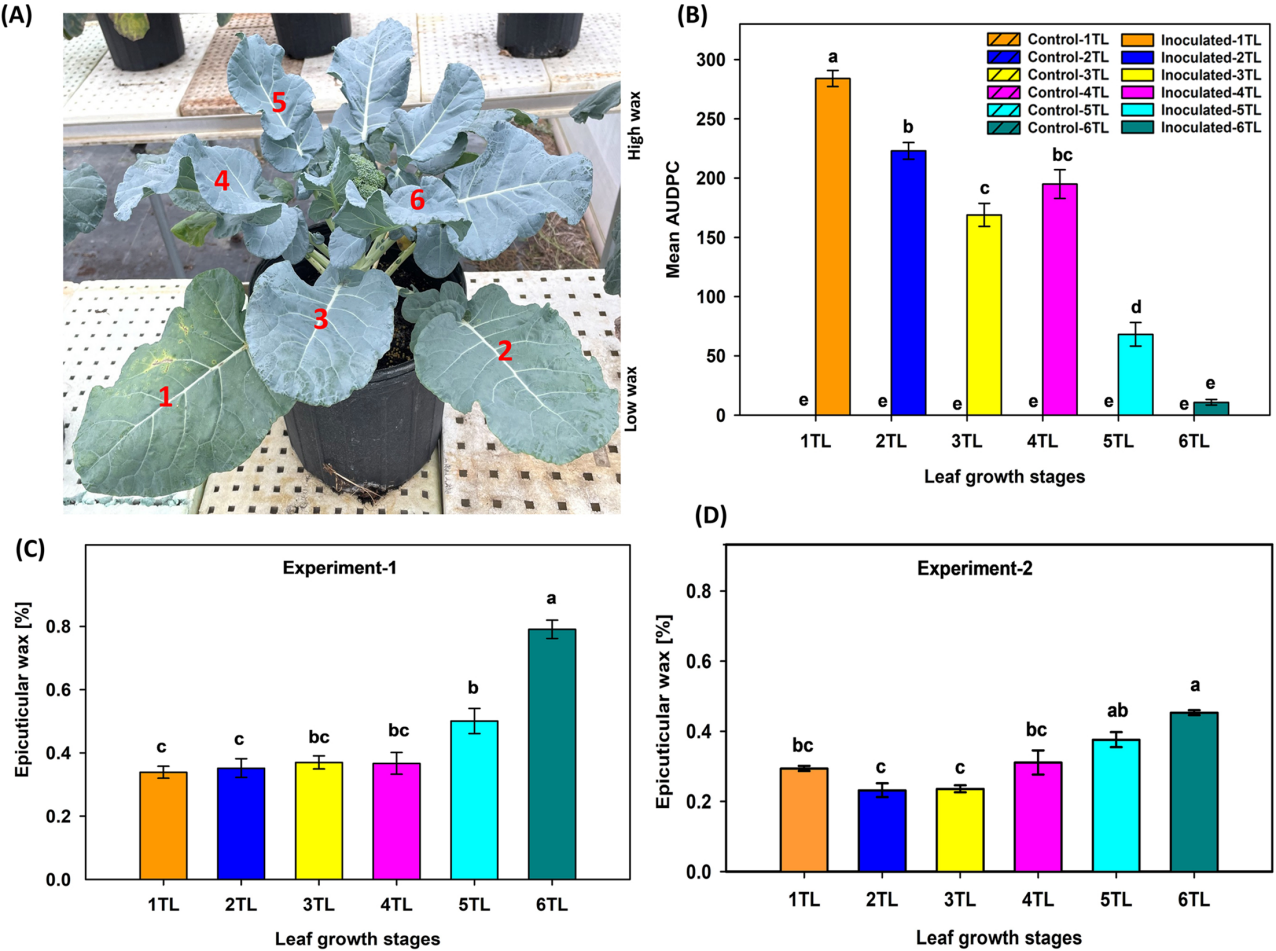
**Variation in age‐related susceptibility and differential epicuticular wax deposition at various leaf growth stages of an intact broccoli plant**. (A) A fully matured broccoli plant with 6–7 true leaves along with the head was inoculated with an aggressive isolate of *A. brassicicola* under greenhouse conditions. Inoculation was done by spraying a spore suspension with a highly aggressive isolate of *A. brassicicola* spore suspension of 10^5^ conidia ml^−1^ until run off. Inoculated leaves were screened for percent disease severity in terms of lesion area (mm^2^) using a severity rating scale of 0 (no necrotic lesions observed) to 100% (entire leaf area is covered with necrotic lesions) at 0, 5‐, 10‐ and 15 DPI. Plants inoculated with sterile water served as a negative control. The panel (A) indicates intact broccoli plants showing the six‐ true paired leaves, where 1TL is at the bottom and 6TL (youngest) is at the top. (B) Bar graphs indicating mean AUDPC values on leaves at different growth stages 1TL (oldest) to 6TL (youngest) of an intact broccoli plant. The panels (C) and (D) represent bar graphs for percent epicuticular wax per unit area on each true leaf of intact broccoli plants during experiment 1 and experiment 2, respectively. Means with same letters are not significantly different according to Tukey's honest significant difference (*p* < 0.05). Epicuticular wax (%) was calculated by extracting the total wax using a chloroform extraction method and the percent wax was calculated [wax (%) = wax weight (mg) /leaf weight (mg) × 100]. The experiments were repeated (Experiment 1 and Experiment 2) independently, with three replicates or plants in each experiment.

In a whole‐plant *in vivo* assessment of leaf‐growth stage mediated susceptibility to *A. brassicicola*, similar observations were made as in the detached leaf assay (Figure 2A). The AUDPC values for the youngest‐top‐most leaf (6TL) was significantly lower than the bottom older leaves (Figure 2B). Interestingly, the bottom most‐oldest leaf (1 TL) had the highest AUDPC value compared with the upper leaves (2TL to 6TL).

The epicuticular wax percentage for leaves at different growth stages were assessed. Since the effect of two experiments on epicuticular wax percentage was significant, each experiment was analyzed and displayed separately in this manuscript. In experiment 1, significantly higher epicuticular wax (%) was observed in the youngest‐top‐most leaf (6TL) compared with the older leaves at the bottom (5 TL to 1 TL; Figure 2C). Similar observations were also made in experiment 2 where significantly higher epicuticular wax (%) was observed in the youngest leaf at the top (6TL) compared to those that were older and at the bottom (5TL to 1TL; Figure 2D).

**FIGURE 3:**
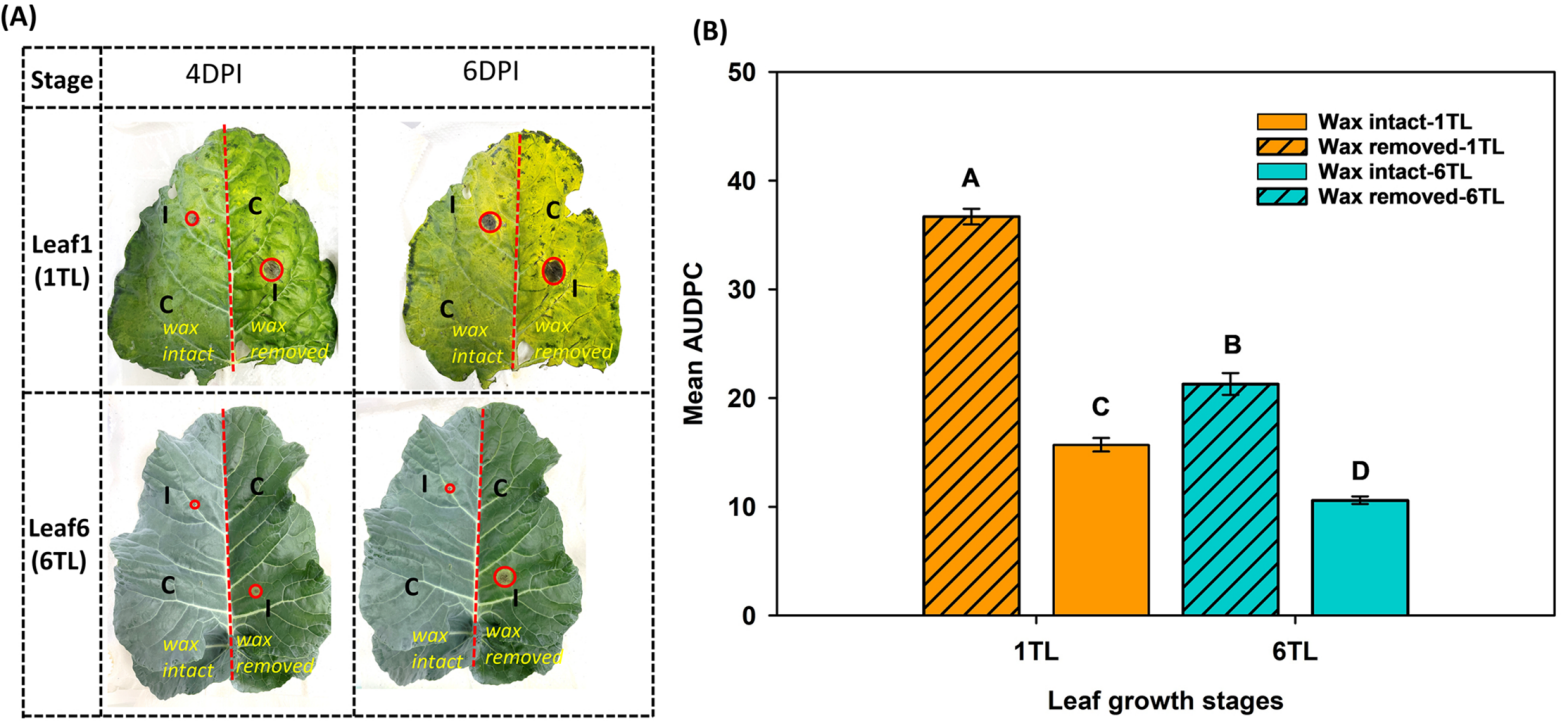
**Pathogenicity assay on youngest (true leaf or TL 6) and oldest leaf (true leaf or TL1) of broccoli with or without the wax layer**. (A) The wax on the leaf surface was removed gently with a cotton plug from the longitudinal half of the leaf surface (right side) divided by the mid‐vein, whereas the other longitudinal half, with an intact wax layer, served as the control. Both surfaces (waxed and non‐waxed) were either inoculated with a highly aggressive isolate of *A. brassicicola* (10 μL of spore suspension of 10^5^ conidia ml^−1^) or sterile water (control). The inoculation sites were marked for fungal inoculation as (I) or control (C) on waxed and non‐waxed leaf surfaces. Leaves were incubated at 24°C at 12 h light and 12 h dark cycle for six days and the lesion areas (mm^2^) on waxed and non‐wax leaf surfaces were recorded at 0, 2, 4 and 6 DPI. (B) represents mean AUDPC values derived from lesion area developed on leaf surfaces of 1 TL or 6TLwith or without epicuticular wax. The experiment was repeated independently with three replicates or leaves for each growth stage in each experiment. Means with the same letters are not significantly different according to Tukey's honest significant difference (*p* < 0.05).

To assess the importance of epicuticular wax, wax layer from one‐half of the leaf was removed and disease severity was assessed upon *A. brassicicola* infection. The removal of epicuticular wax increased the lesion area (Figure 3A) as evident with increased AUDPC values for both leaves at two different growth stages (1TL and 6TL) compared to their other halves with intact epicuticular wax layers at 0, 2, 4 and 6 DPI (Figure 3B).

### 3.2 Relationship between pathogen susceptibility and epicuticular wax percentage

**FIGURE 4:**
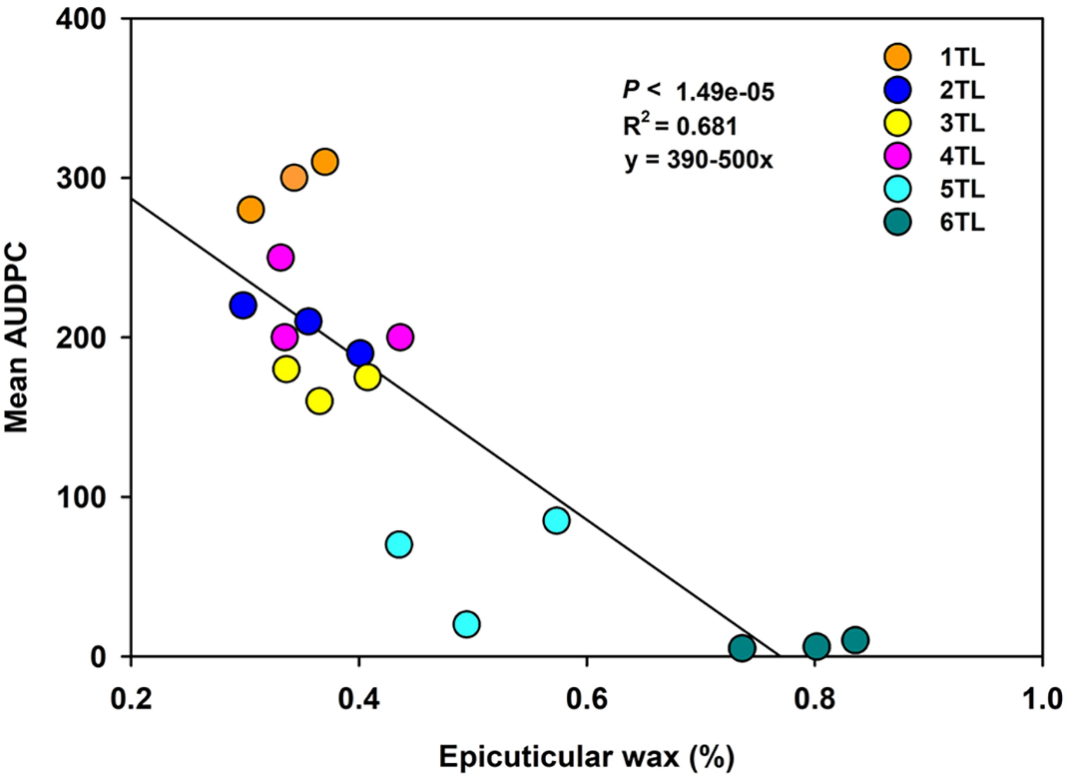
**Regression of percent wax deposition on leaves (%) versus the mean area under disease progress curve (AUDPC)**. Mean AUDPC was calculated from percent disease severity in terms of lesion area (mm^2^) using a severity rating scale of 0 (no necrotic lesions observed) to 100% (entire leaf area is covered with necrotic lesions) at 0, 5, 10 and 15 DPI. Data points represent the mean lesion area (mm^2^) at each sampling period.

Epicuticular wax (%) on the leaf surface of a total of 6 true leaf stages (1 TL to 6 TL) ranged between 0.339–0.791%. The lowest wax deposition was recorded on an oldest bottom‐most leaf (1TL; 0.339%), whereas the highest wax (%) deposition was observed with the youngest top‐most leaf (6TL). Significant differences in epicuticular wax deposition (%) for 2TL (0.351%), 3TL (0.369%) and 4TL (0.367%) were not significantly different from each other. When AUDPC values were calculated upon *A. brassicicola* infection at 2, 4 and 6 DPI, a significantly higher AUDPC value was observed with the oldest bottom‐most leaf (1 TL) compared with the youngest top‐most leaf (6 TL). Interestingly, a negative significant relationship was observed between mean AUDPC and epicuticular wax (%) for the six true leaves (*p* < 1.49 × 10^−5^, R^2^ = 0.681, y = 390‐500x; Figure 4).

### 3.3 Epicuticular wax biosynthetic genes are functional in youngest leaves (6 TL)

**FIGURE 5:**
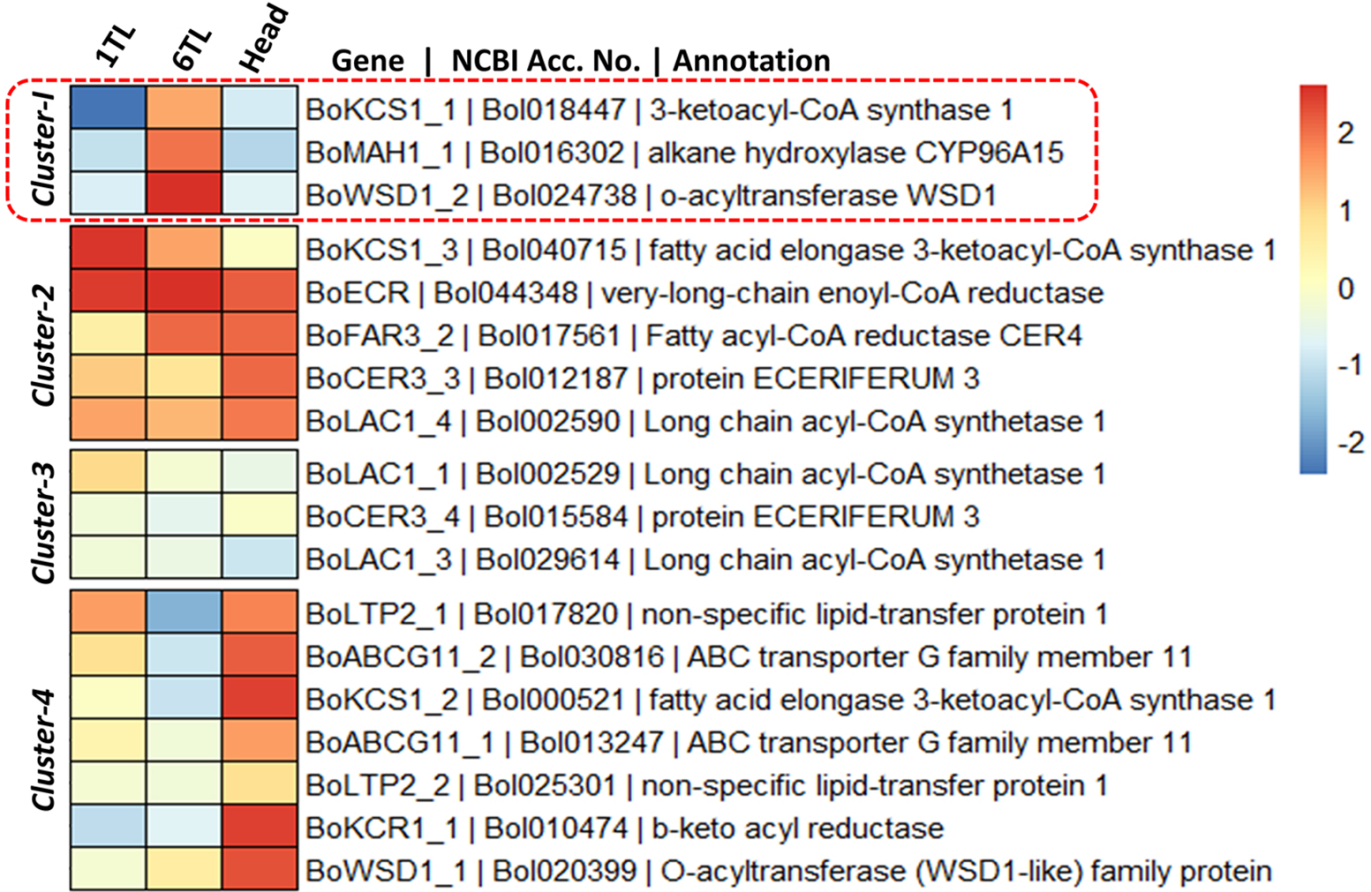
**Mean relative expression of wax biosynthesis genes in 6TL (youngest), 1TL (oldest) leaf and broccoli head**. The Ct (cycle threshold) values for each gene was normalized (ΔCt) using the *actin2* gene as an internal control. The Log transformed values of Sqrt(ΔCt) were used to plot the bi‐color (red and blue) heatmap where dark red and dark blue indicate upregulation and down regulation of genes, respectively.

At the heading, differential gene expression analysis was conducted to compare the transcript level differences for a total of 20 epicuticular wax biosynthesis genes in the youngest leaf at the top (6TL) vs. the oldest leaf at the bottom (1TL) vs. the head of the broccoli plant. Of the 20 wax biosynthesis genes, 18 genes were amplified successfully. Based on expression patterns, the wax biosynthesis genes were grouped into four clusters. In cluster‐1, three genes were grouped together, showing relatively higher expression in the youngest leaf (6TL) as compared to the oldest leaf (1 TL) and head. The genes in cluster‐I included 3‐ketoacyl‐CoA synthase 1 (*Bol018447*), alkane hydroxylase CYP96A15 (*Bol016302*) and O‐acyltransferase WSD1 (*Bol024738*; Figure 5).

In cluster‐2, all genes were equally upregulated in all three tissues at different growth stages (1 TL, 6 TL and head). Cluster‐2 included five genes, namely fatty acid elongase 3‐ketoacyl‐CoA synthase 1 (*Bol040715*), very‐long‐chain enoyl‐CoA reductase (*Bol044348*), fatty acyl‐CoA reductase CER4 (*Bol017561*), protein ECERIFERUM 3 (*Bol012187*), and long chain acyl‐CoA synthase 1 (*Bol002590*; Figure 5). The expression of genes in cluster‐3 was unpredictable and did not show significant expression in either of the three tissues. Three genes were grouped in cluster‐3, including long chain acyl‐CoA synthetase 1 (*Bol002529*), protein ECERIFERUM 3 (*Bol015584*), and long chain acyl‐CoA synthetase 1 (*Bol029614*). A total of seven genes showed upregulation in broccoli heads grouped in cluster 4. Genes in cluster 4 included non‐specific lipid‐transfer protein 1 (*Bol017820*; *Bol025301*), ABC transporter G family member 11 (*Bol030816*; *Bol013247*), fatty acid elongase 3‐ketoacyl‐CoA synthase 1 (*Bol000521*), b‐ketoacyl reductase (*Bol010474*) and O‐acyltransferase (WSD‐like) family protein (*Bol020399*; Figure 5).

### 3.4 *A. brassicicola* infection triggered production of plant hormones in broccoli

**FIGURE 6:**
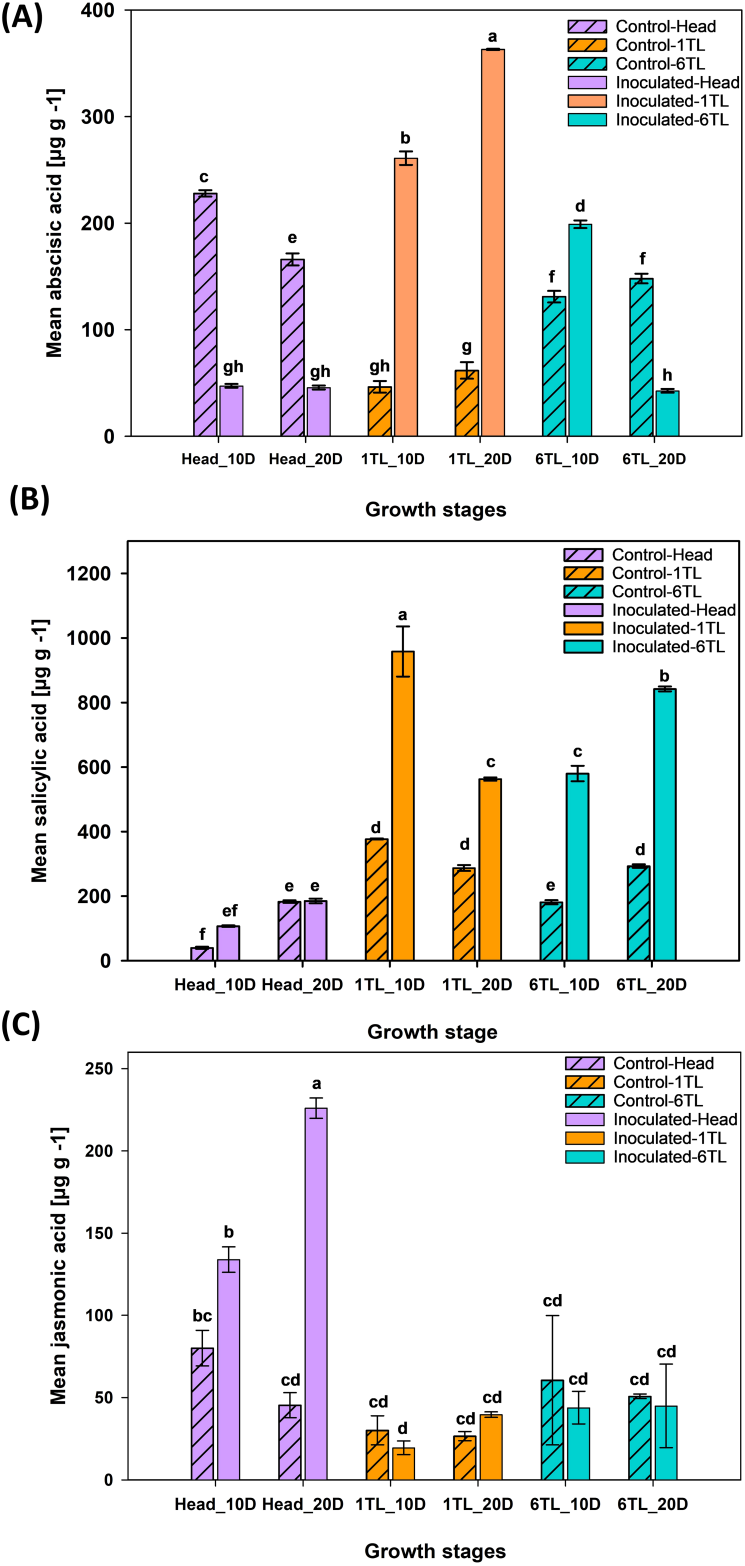
**Estimation of plant hormones in leaves with different ages 6TL (youngest) and 1TL (oldest) leaf and the head of broccoli upon *Alternaria brassicicola* inoculation**. At the head stage, the whole plant was inoculated with a highly aggressive isolate of *A. brassicicola* spore suspension of 10^5^ conidia ml^−1^ or sterile water (control) under greenhouse conditions. The plant tissues [leaves (1 TL and 6 TL) and heads)] of control and inoculated plants were collected at 10 DPI and 20 DPI. Mean concentrations of three plant hormones, abscisic acid (A), salicylic acid (B), jasmonic acid (C), were determined from the leaf and head samples collected after either 10 DPI and 20 DPI. Means with the same letters are not significantly different according to Tukey's honest significant difference (*p* < 0.05).

Plants, being sessile organisms, are easily invaded by pathogens as compared to other eukaryotes. Therefore, plants produce secondary metabolites or antimicrobial compounds and induce defense signaling pathways against these pathogens. In this study, we hypothesized that the plant hormones salicylic acid (SA), jasmonic acid (JA), abscisic acid (ABA) could be involved in inducing the defense responses in broccoli leaf tissues against *A. brassicicola* infection. Plant hormone analysis showed significant differences in the levels of SA, JA and ABA in the youngest top‐most leaf (1TL), oldest bottom‐most leaf (6TL) and the head over a sampling period of 10 and 20 DPI. Higher ABA accumulation was observed in the oldest bottom‐most leaf (1TL) as compared to the youngest top‐most leaf (6TL) of inoculated plants. For instance, in the oldest bottom‐most leaf (1TL), at 10 DPI the ABA content was 268.8 μg g^−1^, it was further increased to 362.7 μg g^−1^ by 20 DPI in inoculated plants whereas in the youngest top‐most leaf (6TL) the ABA concentration was reduced to 42.6 μg g^−1^ by 20 DPI. The ABA concentrations in inoculated head tissue did not change significantly from 10 to 20 DPI and were lower than the non‐inoculated control (Figure 6A).

The trends of salicylic acid showed lower accumulation of SA in the inoculated youngest top‐most leaf (6TL) compared with the oldest bottom‐most leaf (1TL). Also, pathogen inoculation resulted in increased accumulation of SA in leaf tissues, but not in head tissues, compared with the non‐inoculation controls. At 10 DPI the SA content in the oldest bottom‐most leaf (1TL) was recorded as 957.9 μg g^−1^ and it was further reduced to 562.5 μg g^−1^ by 20 DPI. In contrast, in case of the youngest top‐most leaf (6TL), the SA content at 10 DPI was 579.6 μg g^−1^, and by 20 DPI the hormone concentration increased significantly to 841.8 μg g^−1^. The SA concentrations in inoculated head tissues did not change significantly from 10 to 20 DPI and were significantly lower than in the leaf tissues at both growth stages (1TL and 6 TL, Figure 6B).

The JA content was significantly increased in inoculated heads as compared to leaf tissues at both growth stages (1 TL and 6 TL; Figure 6C). The JA content was significantly higher in inoculated (133.8 μg g^−1^) vs. non‐inoculated head tissues (80.1 μg g^−1^) at 10DPI (Figure 6C). The accumulation of JA was more striking by 20 DPI where a significantly higher concentration was recorded (225.9 μg g^−1^; Figure 6C). The increased hormone synthesis of JA and SA upon *A. brassicicola* prompted us to evaluate the expression of *PR* and *NPR1* genes triggered by hormones JA and SA (Figure 6).

### 3.5 *A. brassicicola* infection in different plant tissues (leaves and heads) impacts expression of genes related to induced resistance

**FIGURE 7:**
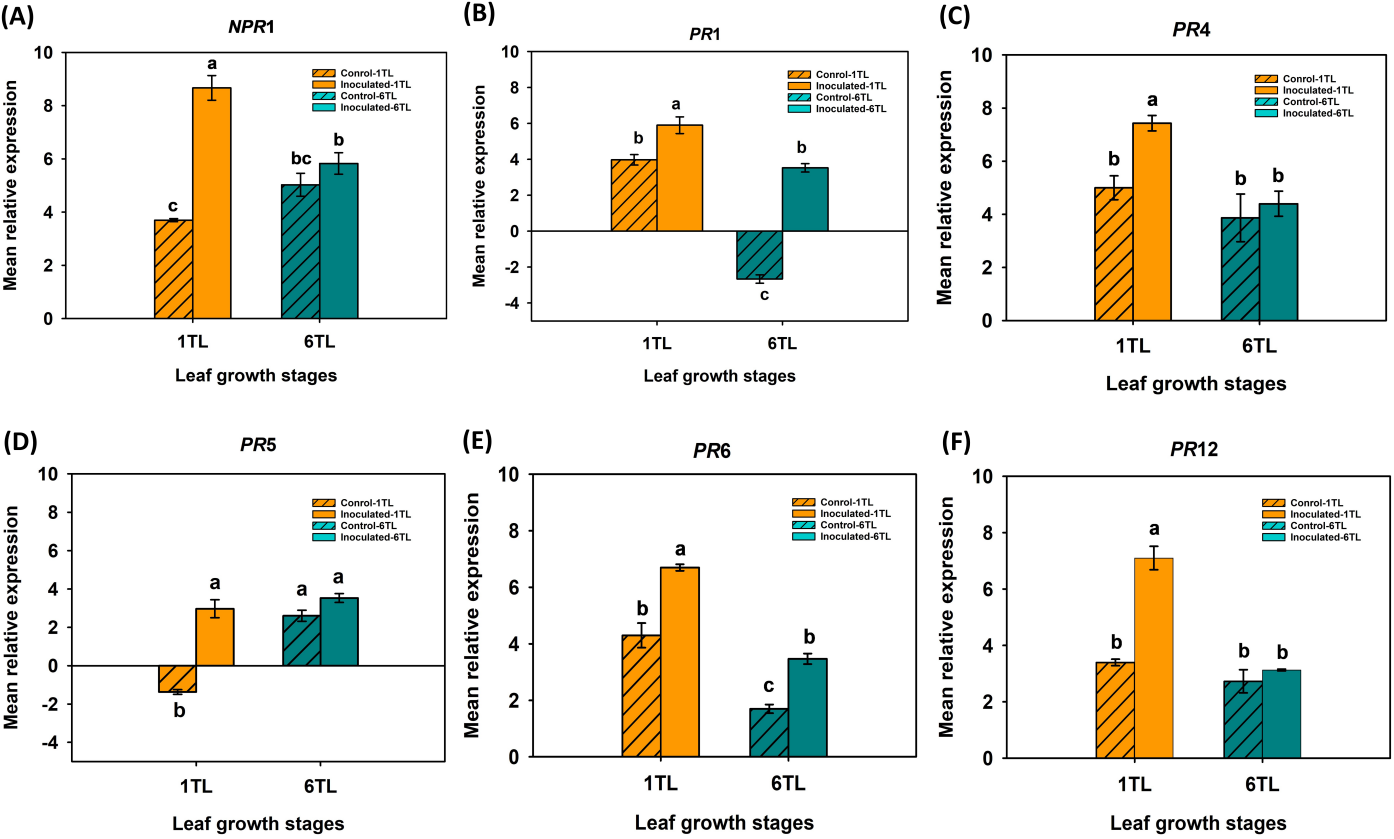
*Alternaria brassicicola* infection in different plant tissues (leaves and heads) impacts expression of genes related to induced resistance. Relative expression of a total of six pathogenesis related genes was analyzed using qRT‐PCR at 10 DPI with a highly aggressive isolate of *A. brassicicola* (10 μL of spore suspension of 10^5^ conidia ml^−1^) or sterile water (control) under greenhouse conditions. The relative expression of PR genes was calculated using normalization with the housekeeping gene *actin2* from the broccoli genome (var. HDEM; GCA_900416815.2). (A) Relative expression of *NPR1*, (B) to (F) indicate the relative expression of *PR1, PR4, PR5, PR6* and *PR12*, respectively. Each bar represents mean relative expression of genes from three replicates in two independent experiments.

Relative expression analysis was carried out for non‐expresser pathogenesis‐related protein 1 (*NPR1*) and pathogenesis‐related (*PR*s) proteins (*PR1, PR4, PR5, PR6* and *PR12*) in the youngest top‐most leaf (6TL) and the oldest bottom‐most leaf (1TL) upon *A. brassicicola* inoculation. The expression of the *NPR1* gene was significantly higher in the oldest bottom‐most leaf (1TL) upon *A. brassicicola* inoculation as compared to the non‐inoculated leaf of the same growth stage. Interestingly, irrespective of the pathogen inoculation, the relative expression of the *NPR1* gene was not significantly different for the youngest top‐most leaf (6TL). Similar observations were made for the genes *PR4*, *PR5*, and *PR12*, where pathogen inoculation did not impact relative expression in the youngest top‐most leaf (6TL). The *PR1*, *PR4*, and *PR6* genes were significantly upregulated in the oldest bottom leaf (1TL) compared to the youngest top‐most leaf (6TL; Figure 7). The *PR12* gene expression was significantly higher in inoculated leaves at 1TL growth stage as compared to its non‐inoculated control. In summary, the *NPR1* and *PR* genes were significantly upregulated and expressed in the oldest bottom‐most leaf (1TL) upon *A. brassicicola* inoculation as compared to the youngest top‐most leaf (6TL; Figure 7).

The relationship between the trends of JA and SA biosynthesis and pathogenesis‐related proteins indicated that the SA level was significantly higher in 1TL. In this context, the expressions of *NPR1*, *PR1*, *PR4*, *PR5*, *PR6*, and *PR12* were notably elevated in inoculated plants compared to their control counterparts. In 6TL, the *PR1* and *PR6* were significantly upregulated in inoculated plants.

### 3.6 Rigorous transcriptome reprogramming in the youngest top‐most leaf (6TL) vs. the oldest bottom‐most leaf (1TL) upon *A. brassicicola* infection

Comparative transcriptome analysis in broccoli plants was conducted between the oldest bottom‐most leaf (1TL) and the youngest top‐most leaf (6TL) when inoculated with *A. brassicicola* or treated with sterile water. A total of 397 million reads were generated for four samples: the oldest bottom‐most leaf (1TL; inoculated and non‐inoculated) and the youngest top‐most leaf (6TL; inoculated and non‐inoculated). Of these, 372 million reads were mapped to the reference genome ‘HDEM’, with an average mapping percentage of 94.1% (Supplementary Table 4).

**FIGURE 8:**
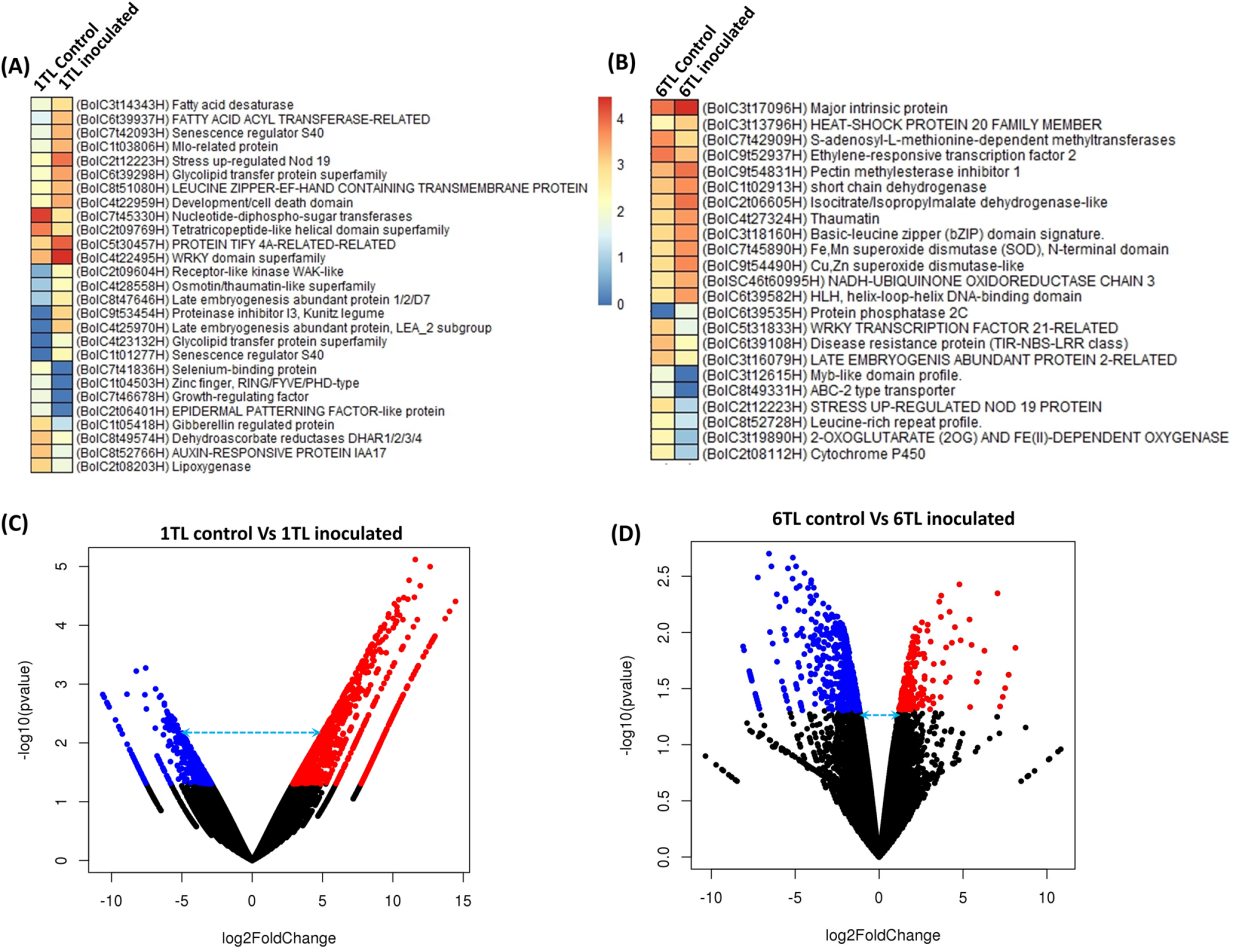
**Transcriptome analysis to identify differentially expressed genes in older and younger leaves of broccoli after *Alternaria brassicicola* inoculation**. (A) Differentially expressed genes (DEGs) between control and inoculated 1TL (older) leaves. (B) DEGs between control and inoculated 6TL (younger) leaves. (C) Volcano plot for DEGs identified between control and inoculated 1TL leaves. (D) Volcano plot for DEGs identified between control and inoculated 6TL leaves. In (C) and (D) the black dots at the bottom indicate genes, which were not significantly expressed in 6TL (younger) and 1TL (older) as compared to their control counterparts. The blue dots indicate significantly downregulated genes. The red dots indicate significantly upregulated genes.

The comparative transcriptome analysis revealed significant differential gene expression between the oldest bottom‐most leaf (1TL) and the youngest top‐most leaf (6TL). In inoculated plants, senescence and stress‐responsive genes were significantly upregulated in the oldest bottom‐most leaf (1TL) compared to the youngest top‐most leaf (6TL). For instance, upregulated genes in the oldest bottom‐most leaf (1TL) included the senescence regulator S40 (*BolC7t42093H*), the stress also upregulated Nod 19 (*BolC2t12223H*), late embryogenesis abundant protein (*BolC8t47646H*) and a WRKY transcription factor (*BolC4t222495H*; Figure 8A; 8C). Conversely, downregulated genes in the oldest bottom‐most leaf (1TL) included a growth‐regulating factor (*BolC7t46678H*), an epidermal patterning factor (*BolC2t06401H*), a gibberellin‐regulated protein (BolC1t05418H), and an auxin‐responsive protein (*BolC8t52766H*). The downregulated genes in the oldest bottom‐most leaf (1TL) included a growth regulating factor (*BolC7t46678H*), an epidermal patterning factor (*BolC2t06401H*), a gibberellin regulated protein (*BolC1t05418H*), and an auxin responsive protein (*BolC8t52766H*).

The genes such as cytochrome P450 (*BolC2t08112H*), leucine rich protein (*BolC8t52728H*), 2‐oxyglutarate and Fe‐dependent oxygenase (*BolC3t19890H*), myb‐like domain protein (*BolC3t12615H*), stress upregulated NOD 19 protein (*BolC2t12223H*), and late embryogenesis abundant protein (*BolC3t16079H*) were significantly down‐regulated in the inoculated youngest top‐most leaf (6TL; Figure 8B; 8D; Supplementary Table 5). In summary, upon *A. brassicicola* infection, the genes associated with stress and senescence were significantly upregulated in the oldest bottom‐most leaf (1TL), while the genes associated with growth‐regulating hormones, such as gibberellin and auxin, were downregulated when compared with the non‐inoculated control plants (Figure 8A; 8C).

## 4 DISCUSSION

The study presented here established the age‐related susceptibility of broccoli foliage to *A. brassicicola*. We demonstrated empirically that older leaves are more susceptible to fungal infection compared with younger leaves. As an example, bottom‐most oldest leaf (1 TL) displayed significantly higher disease severity as evident by larger AUDPCs compared with the youngest top‐most leaf (6 TL) in both the detached leaf and the whole‐plant inoculation assays. Such observations are not common as younger leaves often show significantly higher disease severity than older leaves as in the case of wheat stripe rust caused by *Puccinia striiformis* f. sp. *tritici* (Farber and Mundt, 2017). The authors reported that younger leaves of wheat were more susceptible to *P. striiformis* f. sp. *tritici* than the older leaves.

Our study also elucidated the potential role of epicuticular wax on age‐related susceptibility in broccoli leaves against *A. brassicicola*. We observed a negative relationship between the epicuticular wax percentage and AUDPC across leaves from different growth‐stages. The epicuticular wax percentage increased with decrease in leaf‐age and ‐growth stages that significantly co‐related with an increase in AUDPC in broccoli leaves. These observations partly indicate the decreased susceptibility of younger leaves to *A. brassicicola* with increased accumulation/presence of epicuticular wax. Further removal of epicuticular wax resulted in increased susceptibility to *A. brassicicola*, irrespective of leaf‐growth stages. Leaves with epicuticular wax removed displayed larger lesion areas for both the youngest‐top‐most leaf (6 TL) and the oldest bottom‐most leaf (1 TL). However, when lesion areas induced by pathogen inoculation were compared for the leaves with‐ and without‐epicuticular wax, larger lesions were observed for the later than the former. These observations were consistent irrespective of the leaf‐growth stages, indicating that epicuticular wax may potentially provide a physical barrier to pathogen adhesion, subsequent colonization and lesion area development. Such epicuticular wax mediated resistance/susceptibility has been shown in cauliflower against *A. brassicicola*, where epicuticular wax accumulation facilitated spore‐adhesion and ‐germination with increased lesion area (Berto et al., 1999). Also, in other pathosystems, it was demonstrated that epicuticular wax in sorghum (*Sorghum bicolor*) resulted in the reduced colonization of leaves by *Colletotrichum sublineola*, the causal agent of anthracnose (Xiong et al., 2023). Other than epicuticular wax, glucosinolates are a group of secondary metabolites that have antimicrobial activities contributing to multiple pathogens of Brassica plants including *A. brassicicola* (Saharan et al., 2016). However, there are reports suggesting higher susceptibility to *A. brassicicola* in younger and older leaves despite higher levels of glucosinolates in younger than in older leaves in *Brassica juncea* (Macioszek et al., 2020). The photoperiod also affected necrosis development as considerable necrotic lesions were formed on *B. juncea* grown under a 16 h day/8 h night photoperiod at 72 hours post‐inoculation (hpi; Macioszek et al., 2021).

Interestingly, the differences in epicuticular wax percentages among leaves belonging to closely related growth stages that occurred between the youngest top (6 TL) and the oldest bottom (leaf 1) (5 TL to 2 TL) were not significantly different and also did not display significant differences in their susceptibility to *A. brassicicola*. The only striking difference in epicuticular wax and AUDPC was observed with the oldest bottom (1 TL) and the youngest top (6 TL) leaves. These observations suggest that epicuticular wax content does not seem to vary drastically among leaves at closer growth stages with similar levels of susceptibility to *A. brassicicola*. However, these observations may vary with different broccoli genotypes and environmental conditions, which can be assessed in the future.

Plant surfaces are exposed to various environmental stresses, a layer of cuticular wax on aerial parts acts as a physical barrier and protects plant tissues from enormous biotic and abiotic stresses such as excessive transpiration, UV radiation and pathogen infections (Xue et al., 2017; Dimopoulos et al., 2020; Wan et al., 2020; Klavins and Klavins, 2020; Yuan et al., 2020). It has been established that thicker cuticular wax inhibits the wetting of the leaf surface due to its glossiness and impermeability, which limits solubilization and diffusion of nutrients from the leaf tissue providing resistance to pathogen pre‐penetration and infection (Bodenhausen et al., 2014; Lindow and Brandl, 2003; Schlechter et al., 2019).

Our study identified three key epicuticular wax biosynthesis genes, 3‐ketoacyl‐CoA synthase 1 (*Bol018447*), alkane hydroxylase CYP96A15 (*Bol016302*) and O‐acyltransferase WSD1 (*Bol024738*). The 3‐ketoacyl‐CoA synthase (KCS) enzymes govern the rate‐limiting step in the production of very‐long‐chain fatty acids (VLCFAs), thus playing a crucial role in regulating wax biosynthesis (Yang et al., 2021). The cytochrome P450 enzyme CYP96A15, also referred to as midchain alkane hydroxylase (MAH1), is crucial in plant wax biosynthesis. It catalyzes the conversion of alkanes into secondary alcohols and ketones through hydroxylation on the leaf surface (Greer et al., 2007). The O‐acyltransferase WSD1 is a bifunctional enzyme that catalyzes the synthesis of wax esters. Wax esters are a component of cuticular wax, a hydrophobic layer, which protects plants from biotic and abiotic stresses (Li et al., 2008). Higher expression of these key epicuticular wax biosynthesis genes supports higher levels of epicuticular wax and corresponding lower susceptibility to *A. brassicicola* in younger leaves (6 TL) compared with older leaves (1 TL).

Our findings also demonstrated an increased accumulation of ABA in older leaves (1 TL) in response to *A. brassicicola* infection, which could be responsible for increased senescence. These observations are quite common in fields where the senescence of older leaves occurs with *A. brassicicola* infections. In contrast, the ABA levels decreased in younger leaves (6 TL), which remained intact and considerably fewer symptoms of senescence (yellowing) were observed. Interestingly, the ABA levels were substantially decreased in the head tissue after *A. brassicicola* inoculation compared to the non‐inoculated controls. Generally, ABA is involved in the plant defense against abiotic stresses, especially drought. The basal levels estimated with non‐inoculated plants were significantly lower in leaves at the 1 TL stage as compared to leaves at the 6 TL stage, while the broccoli heads had the highest concentration. The trend was the opposite when the plants were challenged with *A. brassicicola*; the concentration of ABA increased 5‐fold in leaves at 1 TL as compared to leaves at 6 TL. Interestingly, the broccoli heads had significantly lower concentrations of ABA in inoculated plants and it did not change as the plant aged (10 DPI to 20 DPI). It seems *A. brassicicola* inoculation resulted in modulation of ABA hormone concentrations in broccoli head and potentially resulted in increased susceptibility to infection. It is interesting as researchers previously reported that endogenous level of ABA is a key determinant factor for the susceptibility of *Arabidopsis thaliana* against *Meloidogyne paranaensis* (Yop et al., 2023). However, in our case endogenous or basal levels of ABA did not directly impact *A. brassicicola*. Future research on the role of ABA and the components of ABA signaling against *A. brassicicola* in broccoli needs to be further evaluated.

Pathogen inoculation resulted in higher accumulation of SA across leaf‐ (1TL and 6 TL) and head‐ tissues compared to non‐inoculated controls. These observations were not uncommon as pathogen infection results in accumulation of SA in tissues and induces SAR that protects the plant from subsequent attacks (Lefevere et al., 2020; Kim and Hwang, 2014; Zhang and Li, 2019). The accumulation of SA activates expression of NPR and PR genes and induces the SAR against pathogens. Overexpression of *NPR1* in *A. thaliana* leads to an enhanced broad‐spectrum resistance to the pathogens *Pseudomonas syringae* and *Peronospora parasitica* with no adverse effects on the plant (Cao et al., 1998). Our results indicated a trend where *NPR1* and *PR* genes were significantly upregulated and expressed in the oldest bottom‐most leaf (1 TL) upon *A. brassicicola* inoculation as compared to the youngest top‐most leaf (6 TL) indicating a potential induction of SAR. However, pathogen infection was significantly severe in the former than the later, which was contrary to observations made in other pathosystems (Pieterse et al., 1998, Dutta et al., 2017). It is possible that SAR induction in oldest bottom‐most leaf (1 TL) might not be potent to protect these leaf tissues from *A. brassicicola*, which is an aggressive pathogen with presumed partial necrotrophic phase.

With regards to JA, pathogen inoculation did not change the JA accumulation in both leaf tissues (1 TL and 6 TL) compared to the non‐inoculated controls. In contrast, JA accumulation significantly increased in head after *A. brassicicola* infection compared to other leaf tissues (1 TL and 6 TL) indicating potential induction of JA‐mediated SAR in head tissues. However, it was not the case as severe infection were observed on head tissues too. Future research on the role of JA and the components of JA signaling against *A. brassicicola* in broccoli need to be further evaluated.

Using transcriptomics, we observed that *A. brassicicola* infection triggered a large number of senescence related genes in the oldest bottom leaves (1TL), which is not surprising as more often older leaves display yellowing and enhanced senescence when infected with fungal pathogens. The oldest bottom leaves (1TL) also showed a large number of genes significantly downregulated or upregulated in stress‐response to pathogen inoculation indicating potential alteration of genes related to host‐pathogen interactions in broccoli against *A. brassicicola*. Further investigations on different plant‐defense and stress‐response pathways in broccoli against *A. brassicicola* in different tissues need to be evaluated.

We also observed a number of genes upregulated or downregulated in the wax biosynthesis pathway in leaf tissues from the oldest bottom leaves (1 TL) upon *A. brassicicola* inoculation. These observations indicate that apart from the lower accumulation of epicuticular wax in the oldest bottom leaves (1 TL) compared to the youngest top‐most leaf (6 TL), *A. brassicicola* infection may also impact its accumulation resulting severe infection. Similar findings were reported earlier in pea where authors showed the influence of the physical structure and the chemical composition of epicuticular leaf waxes, on the pre‐penetration processes of biotrophic fungi, *Erysiphe pisi* (a causal agent of powdery mildew in pea). The authors demonstrated that the germination rates of *E. pisi* were higher on the adaxial surface with lower accumulation of epicuticular wax compared to the abaxial surface (Gniwotta et al., 2005). Among the important components of epicuticular wax, very long chain fatty acids (VLCFAs) are a major component, which are the outermost layer of plant cuticles (Jetter et al., 2006). Reports suggest that the C26 aldehydes in VLCFAs are responsible for the triggering of *Blumeria graminis* f. sp. *hordei* pre‐infection structures (Hansjakob et al., 2010, Hansjakob et al., 2011, Hansjakob et al., 2012). Therefore, the composition of cuticular wax and VLCFAs may potentially determine the outcome of host‐pathogen interaction as it affects the process of penetration and infection (Lewandowska et al., 2020). It would be interesting to assess if VLCFAs in epicuticular wax play an important role in colonization of *A. brassicicola* on broccoli in different developmental stages of plant growth. Further studies should focus on specific components of VCLFAs that may influence *A. brassicicola* infection in broccoli.

## AUTHOR CONTRIBUTIONS

S.S.G. and B.D. conceived the idea and designed the experiments. B.D. acquired the funding and provided resources to accomplish all experiments. S.S.G. performed all experiments and RNA‐Seq analysis. N.K. contributed to experimentation and statistical data analysis. S.S.G. wrote original draft. N.K., B.G., B.D., S.S.G. revised the draft and approved final version of manuscript.

## FUNDING INFORMATION

This project was funded by USDA NIFA SCRI (2020–51181‐32062).

## CONFLICT OF INTEREST STATEMENT

All authors declare that there no potential conflicts of interest.

## Supporting information


**Data S1:** Tables.

## Data Availability

The sequencing data generated for RNA‐Seq samples in deposited in Zenodo public data repository (https://zenodo.org) with records https://zenodo.org/records/14062447 for top control and top inoculated samples. Similarly, the sequencing data for bottom control and inoculated samples is deposited at https://doi.org/10.5281/zenodo.14061431.
